# Supplementary material for: Resolving the full spectrum of human genome variation using Linked-Reads
Source: Genome Res. 2019 Apr;29(4):635–45. doi: 10.1101/gr.234443.118 (PMC6442396; doi:10.1101/gr.234443.118)
Supplement: Supplemental Material [file supp_gr.234443.118_Supplemental_longranger-2.2.zip › longranger-2.2.tar.bz2!/lr2.2-public/lib/stan/cmdstan/stan/doc/api/html/math_2functions_2is__nan_8hpp_source.html]

Stan: src/stan/math/functions/is\_nan.hpp Source File


|  |  |
| --- | --- |
|  | Stan  2.5.0  probability, sampling & optimization |


- Main Page
- Related Pages
- Namespaces
- Classes
- Files

- File List
- File Members

All Classes Namespaces Files Functions Variables Typedefs Enumerations Enumerator Friends Macros Pages

- src
- stan
- math
- functions

is\_nan.hpp

Go to the documentation of this file.

1 #ifndef STAN\_\_MATH\_\_FUNCTIONS\_\_IS\_NAN\_HPP

2 #define STAN\_\_MATH\_\_FUNCTIONS\_\_IS\_NAN\_HPP

3

4 #include <boost/math/special\_functions/fpclassify.hpp>

5

6 namespace stan {

7

8  namespace math {

9

18  inline int

19  is\_nan(const double x) {

20  return boost::math::isnan(x);

21  }

22

23  }

24 }

25

26 #endif

boost::math::isnan

bool isnan(const stan::agrad::var &v)

Checks if the given number is NaN.

**Definition:** boost\_fpclassify.hpp:69

stan::math::is\_nan

int is\_nan(const double x)

Returns 1 if the input is NaN and 0 otherwise.

**Definition:** is\_nan.hpp:19


---

[ Stan Home Page ]

*© 2011–2014,
Stan Development Team.*
